# From Spent Black and Green Tea to Potential Health Boosters: Optimization of Polyphenol Extraction and Assessment of Their Antioxidant and Antibacterial Activities

**DOI:** 10.3390/antiox13121588

**Published:** 2024-12-23

**Authors:** Ahlam Harfoush, Aseel Swaidan, Salma Khazaal, Elie Salem Sokhn, Nabil Grimi, Espérance Debs, Nicolas Louka, Nada El Darra

**Affiliations:** 1 Department of Nutrition and Dietetics, Faculty of Health Sciences, Beirut Arab University, Tarik El Jedidah, Riad El Solh, P.O. Box 115020, Beirut 1107 2809, Lebanon; ahlam.harfoush@bau.edu.lb (A.H.); ais489@student.bau.edu.lb (A.S.); s.khazaal@bau.edu.lb (S.K.); n.aldarra@bau.edu.lb (N.E.D.); 2 Department of Medical Laboratory Technology, Faculty of Health Sciences, Beirut Arab University, Beirut 1107 2809, Lebanon; e.sokhn@bau.edu.lb; 3 Centre de Recherche Royallieu-CS 60319, ESCOM, TIMR (Integrated Transformations of Renewable Matter), Université de Technologie de Compiègne, 60203 Compiègne CEDEX, France; 4 Department of Biology, Faculty of Arts and Sciences, University of Balamand, P.O. Box 100, Tripoli 1300, Lebanon; esperance.debs@balamand.edu.lb; 5 Unité de Recherche Technologies et Valorisation Agro-Alimentaire, Centre d’Analyses et de Recherche, Faculté des Sciences, Université Saint-Joseph de Beyrouth, Mar Roukos—Dekwaneh, Riad El Solh, P.O. Box 1514, Beirut 1107 2050, Lebanon; nicolas.louka@usj.edu.lb

**Keywords:** spent black tea, spent green tea, polyphenols, antioxidant, antibacterial, waste management

## Abstract

Tea, one of the most popular beverages worldwide, generates a substantial amount of spent leaves, often directly discarded although they may still contain valuable compounds. This study aims to optimize the extraction of polyphenols from spent black tea (SBT) and spent green tea (SGT) leaves while also exploring their antioxidant and antibacterial properties. Response surface methodology was utilized to determine the optimal experimental conditions for extracting polyphenols from SBT and SGT. The total phenolic content (TPC) was quantified using the Folin–Ciocalteu method, while antioxidant activity was evaluated through the DPPH assay. Antibacterial activity was assessed using the disk diffusion method. Additionally, high-performance liquid chromatography (HPLC) was employed to analyze the phytochemical profiles of the SBT and SGT extracts. Optimal extraction for SBT achieved 404 mg GAE/g DM TPC and 51.5% DPPH inhibition at 93.64 °C, 79.9 min, and 59.4% ethanol–water. For SGT, conditions of 93.63 °C, 81.7 min, and 53.2% ethanol–water yielded 452 mg GAE/g DM TPC and 78.3% DPPH inhibition. Both tea extracts exhibited antibacterial activity against Gram-positive bacteria, with SGT showing greater efficacy against *S. aureus* and slightly better inhibition of *B. subtilis* compared to SBT. No activity was observed against the Gram-negative bacteria *E. coli* and *S. typhimurium*. HPLC analysis revealed hydroxybenzoic acid as the main phenolic compound in SBT (360.7 mg/L), while rutin was predominant in SGT (42.73 mg/L). The optimized phenolic-rich extracts of SBT and SGT demonstrated promising antioxidant and antibacterial potential, making them strong candidates for use as natural health boosters in food products.

## 1. Introduction

Tea, derived from the *Camellia sinensis* Linn plant, is one of the most widely consumed beverages worldwide. Its history dates back 5000 years in China, where it was initially utilized for medicinal purposes [1]. Over recent decades, tea consumption has significantly increased due to its health benefits and refreshing taste. According to the Food and Agriculture Organization (FAO), two-thirds of the world’s population regularly drink tea, making it a staple beverage [2].

Tea is categorized into four main types based on its processing and fermentation levels: black, green, oolong, and white. Each type is produced using distinct methods. For instance, black tea is fully fermented, allowing enzyme-mediated oxidation to occur, whereas green tea undergoes minimal processing to prevent polyphenol oxidation [3]. Among these types, black tea is the most popular, followed by green, oolong, and white teas [1].

The health benefits of tea are largely attributed to its polyphenolic content, which makes up 10–30% of the dry leaf weight. Polyphenols include catechins, theaflavins, flavanols, and phenolic acids, with gallic acid being the predominant phenolic acid [4]. They are responsible for tea’s antioxidant, antimicrobial, and anti-inflammatory properties. Several studies have examined the phenolic content and composition of tea, demonstrating the variability of these compounds according to several factors, including tea type, season, origin, fermentation degree, and extraction method [5]. As a non-fermented beverage, green tea is considered to have a higher concentration of polyphenols [6,7], contributing to its greater antioxidant potential compared to black tea [8,9].

Various extraction techniques have been used to isolate bioactive compounds from tea leaves. While non-conventional methods, such as pulsed electric field extraction and ultrasound-assisted extraction, have shown higher yields [10], the conventional water bath extraction method remains the most widely used due to its simplicity and affordability [11]. Solvent choice, extraction time, temperature, and the solid-to-solvent ratio can significantly influence the extraction yield [12]. Combining water with organic solvents, such as ethanol and methanol, has been shown to enhance the yield and solubility of compounds, while reducing the extraction time [13,14,15]. Response surface methodology (RSM) is often utilized to optimize the process parameters for extracting phytochemicals [16].

Extensive research has focused on the health benefits of fresh tea leaves, particularly their antioxidant and anti-inflammatory properties [17,18,19,20,21]. Studies have shown that tea polyphenols effectively scavenge free radicals and reactive oxygen species produced by various forms of oxidative stress [22,23]. In addition, studies suggest that regular tea consumption may reduce the risk of cancers, including stomach, colon, and pancreas [24,25,26]. Furthermore, black and green tea have demonstrated antibacterial properties, particularly against *Staphylococcus aureus* and *Bacillus cereus* [22,27]. An inverse correlation exists between the antibacterial activities of tea and the level of fermentation, indicating that green tea exhibits stronger antibacterial properties than black tea [28,29].

The daily consumption of tea generates a significant amount of spent leaves, which are often discarded as waste. This disposal not only contributes to environmental burdens, but also leads to the loss of both spent black tea (SBT) and spent green tea (SGT), which may still contain bioactive compounds suitable for repurposing in research or industrial applications [30]. Given the documented health benefits and biological activities of polyphenols, exploring the valorization of polyphenol-rich food waste represents a significant step toward reducing environmental pollution. This approach also highlights the potential for incorporating bioactive compound-rich by-products into food products or supplements. Although several studies have focused on spent tea leaves [30,31,32,33,34], this study uniquely focuses on optimizing the extraction conditions (time, temperature, and ethanol-to-water ratio) to maximize the total phenolic content and antioxidant activity of both the SBT and SGT extracts. Furthermore, the antibacterial properties and phenolic profiles of these optimized extracts are evaluated. This dual approach of optimization and functional evaluation highlights the novelty of this work, as it not only enhances the valorization of spent tea leaves but also explores their potential as natural antioxidants and antibacterial agents.

## 2. Materials and Methods

### 2.1. Raw Material

**Figure 1:**
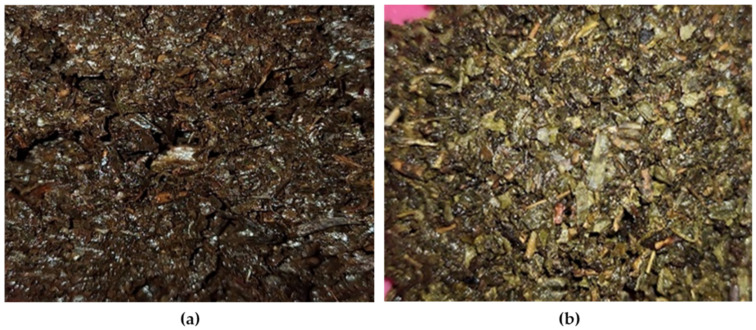
Tea samples: (**a**) spent black tea (SBT); (**b**) spent green tea (SGT).

Bulk samples of the most common brands of black and green tea in Lebanon were purchased. The black and green teas originated from Sri Lanka (also known as Ceylon). To ensure quality and homogeneity, the tea samples were freshly prepared in our lab, rather than sourcing pre-used leaves, which could potentially have variable origins and contamination. The preparation procedures for black and green tea were developed by screening tea-making methods at a ‘Salon du Thé’ and local cafeterias in Lebanon, ensuring alignment with consumer preferences. Specifically, 20 g of black tea leaves was steeped in 2370 mL of hot water at 74 °C for 40 s, and green tea leaves at 80 °C for 2 min. After brewing, the tea leaves were pressed through a strainer (Figure S1), rather than merely dipped and removed, to ensure maximum removal of tea liquid from the spent tea leaves and to better simulate natural infusion processes. The pressed tea leaves were collected as SBT and SGT (Figure 1) for further analyses.

### 2.2. Chemicals, Reagents, and Media

All chemicals and reagents used for this study were of analytical grade. Folin–Ciocalteu reagent, sodium carbonate (Na_2_CO_3_), gallic acid (3,4,5-trihydroxybenzoic acid), 2,2-diphenyl-picrylhydrazyl (DPPH), Trolox (6-hydroxy-2,5,7,8-tetramethylchromane-2-carboxylic acid), and HPLC standards were purchased from Sigma-Aldrich (Steinheim, Germany). Mueller–Hinton Agar (MHA) and Mueller–Hinton Broth (MHB) were acquired from HIMEDIA (Mumbai, India).

### 2.3. Determination of Dry Matter

The spent tea leaves were placed in a ventilated oven at 105 °C for 24 h to measure the dry matter (DM) content. The DM content was then calculated and expressed as a percentage of the total weight of the leaves. It was determined that the dry matter content of both spent tea leaves was around 26% *w*/*w*.

### 2.4. Extraction Procedure

RSM was employed to optimize the extraction parameters [35]. Water bath extraction (WB) was conducted using a water bath shaker (DKZ-1 series) [33]. Spent tea leaves were placed in a round-bottom flask containing the appropriate concentration of ethanol. The mixture was maintained at the specified temperature for the designated time (Section 2.5) while being connected to a condenser setup to ensure effective extraction. Following extraction, the mixtures were filtered to obtain the filtrates, which were then centrifuged for 10 min at 5000 rpm. The supernatants were then concentrated using a rotary evaporator (Heidolph, Germany) at 40 °C to remove the solvent and were subsequently stored at −20 °C for future analyses.

### 2.5. Experimental Design

In addition to the solid-to-solvent ratio, several other factors can affect both the quantity and quality of the extracted phenolic compounds. To optimize the extraction process, RSM was employed to evaluate the influences of extraction temperature (T), extraction time (t), and the ethanol/water ratio (E/W), as well as the interactions between these parameters. A rotatable central composite design (2^3^ + star) was utilized, which included twenty experimental runs with four replicates at the central points. This design aimed to analyze how temperature, time, and solvent ratio impacted TPC and DPPH activity as response variables. The parameters varied as follows: temperature ranged from 40 to 80 °C, extraction time from 30 to 100 min, and ethanol/water ratio from 30 to 70%. The highest and lowest levels of these factors were coded as +1 and −1, respectively. This experimental design was consistently applied for both SBT and SGT extraction methods.

Considering three parameters, the experimental data were fitted to obtain a second-degree regression equation as follows:

Y = β_0_ + β_1_ × T + β_2_ × t + β_3_ × E/W + β_4_ × T^2^ + β_5_ × T × t + β_6_ × T × E/W + β_7_ × t^2^ + β_8_ × t × E/W + β_9_ × E/W^2^

where “Y” is the predicted response parameter; α_0_ is the mean value of the responses at the central point of the experiment; β_1_, β_2_, and β_3_ are the linear coefficients; β_4_, β_7_, and β_9_ are the quadratic coefficients; and β_5_, β_6_, and β_8_ are the interaction coefficients.

### 2.6. Determination of the TPC

The Folin–Ciocalteu (FC) procedure was employed for the determination of TPC in the spent tea samples according to a method described previously [36]. While the FC reagent reacts with phenolic compounds through oxidation, it is not entirely specific to polyphenols and may also interact with other reducing agents, such as sugars and ascorbic acid. Therefore, the results of the TPC assay reflect the overall reducing capacity of the sample rather than exclusively the phenolic content [37,38]. A volume of 0.5 mL of Folin–Ciocalteu reagent (diluted 1/10, *v*/*v*) was added to 0.1 mL of the sample. Then, 0.4 mL of Na_2_CO_3_ 7.5% (*w*/*v*) was added. The same procedure was used to prepare the blank, except that 0.1 mL of distilled water was used in place of the sample. This was followed by 10 min of incubation at 60 °C, followed by another 10 min at 4 °C. The absorbance was recorded at 750 nm using a UV–Vis spectrophotometer (OPTIMA SP-300; Kanagawa, Japan). Quantification was carried out using a calibration curve, with gallic acid as the standard compound. TPC values were extrapolated from the calibration curve equation and expressed as milligrams of gallic acid equivalents per gram of dry matter (mg GAE/g DM).

### 2.7. Determination of the Antioxidant Activity

The antioxidant activity of the spent tea leaf extracts was evaluated by their ability to scavenge the free radical DPPH [39]. For sample analysis, 1.45 mL of DPPH solution was added to 50 µL of each extract or Trolox (positive control) or methanol (negative control). The absorbance of the resulting mixture was then measured at 515 nm after incubation in the dark at room temperature for 30 min, using pure methanol as a blank. Antioxidant activity was expressed as the percentage of DPPH inhibition according to the following formula:

$$Inhibition\;percentage\;(\%)=\frac{Absorbance\;of\;the\;negative\;control-Absorbance\;of\;the\;sample}{Absorbance\;of\;the\;negative\;control}\times 100$$

### 2.8. High-Performance Liquid Chromatography Analysis

The identification and quantification of phenolic compounds in the SBT and SGT extracts were carried out using high-performance liquid chromatography (HPLC) at the Lebanese Agricultural Research Institute (LARI), following the protocol outlined by Vizzotto et al. (2007) [40]. The utilized HPLC system for this analysis was an Agilent 1100 Series system (Teknokroma Professional Friendly Lichrospher 100 RP18 5 mM, 25 × 0.46, Serial number NF-21378, Barcelona, Spain) equipped with an autosampler, a Zorbax column oven (Barcelona, Spain), and a diode array detector (DAD). To separate phenolic compounds, a C18 column (250 × 4.6 mm; 5 µm) was used. The following standards were used for identification and quantification: gallic acid, protocatechuic acid, hydroxybenzoic acid, catechin, chlorogenic acid, caffeic acid, *p*-coumaric acid, rutin, ellagic acid, trans-cinnamic acid, and quercetin. The mobile phase consisted of acidified purified water with a pH of 2.3 with HCl (A) and methanol (B) of HPLC grade. The elution process was carried out under isocratic conditions, starting with 85% A and 15% B from 0 to 5 min. Then, a gradient profile was applied from 5 to 30 min, transitioning from 85% A and 15% B to 0% A and 100% B. This was followed by isocratic conditions from 30 to 35 min with 0% A and 100% B. The injection volume was 10 µL, and the flow rate was set at 1 mL/min. Phenolic compounds were identified by comparing the retention times of the observed peaks with those of standard compounds. The concentrations of these phenolic compounds were determined by creating standard curves for each specific compound, using different concentrations of corresponding standards.

### 2.9. Evaluation of the Antibacterial Activity

#### 2.9.1. Preparation of the Standardized Inoculum

The antibacterial activity of the spent tea extracts was assessed against four bacterial strains: two Gram-positive bacteria (*Staphylococcus aureus* ATCC 25923 and *Bacillus subtilis* ATCC 6633) and two Gram-negative bacteria (*Salmonella typhimurium* ATCC 14028 and *Escherichia coli* ATCC 25922). Well-isolated and morphologically consistent bacterial colonies were gently transferred to a sterile saline solution using a sterile loop and mixed well, ensuring a homogenous dispersion. The turbidity of the resulting suspension was then compared and adjusted to match the turbidity of a prepared 0.5 McFarland standard (10^8^ CFU/mL) under optimal lighting conditions [41].

#### 2.9.2. Disk Diffusion Assay

The disk diffusion assay was performed according to a method described by Abbaszadegan et al. (2016) [42]. Briefly, the prepared standardized bacterial suspensions were uniformly spread on MHA plates using sterile cotton swabs. Subsequently, sterile 5 mm filter paper disks were aseptically positioned on the agar surface and gently pressed onto the medium using sterile forceps. Each disk received 20 µL of the respective tea extract. Sterile distilled water-impregnated disks served as negative controls, while Gentamicin disks (50 μg) established positive controls. The inoculated plates were incubated at 37 °C for 24 h. After incubation, the diameters of any resulting zones of inhibition surrounding the disks were measured (in millimeters).

### 2.10. Statistical Analysis

All experiments were performed in triplicate to ensure the validity and reproducibility of the results. Data are expressed as mean values ± standard deviations (SDs). The statistical significance was assessed using IBM-SPSS Statistics for Windows, Version 25.0 (Released 2017. IBM Corp., New York, NY, USA), employing one-way ANOVA followed by the least significant difference (LSD) test to compare individual means. *p*-values less than 0.05 were considered statistically significant, indicating a confidence level of over 95%. Data were processed using STATGRAPHICS Centurion XVI.I (Statgraphics 18, The Plains, VA, USA) for the optimization of the extraction process.

## 3. Results and Discussion

### 3.1. Influence of Extraction Time, Temperature, and Ethanol Percentage on TPC Yield and DPPH Inhibition Percentage

RSM was applied to optimize the extraction conditions for polyphenols, maximizing the TPC and antioxidant activity. The model was designed by varying the temperature, extraction time, and E/W ratio while maintaining a solid-to-solvent ratio of 1/25 (g/mL) for SBT and 1/20 (g/mL) for SGT, based on previous findings [26,27]. Table 1 presents the TPC values (mg GAE/g DM) and DPPH inhibition percentages for both extracts across the twenty runs. Among the tested conditions, the highest TPC values were 405 mg GAE/g DM for SBT and 435 mg GAE/g DM for SGT. In terms of antioxidant activity, SBT achieved 50.4% inhibition, while SGT reached 77.2%.

**Figure 2:**
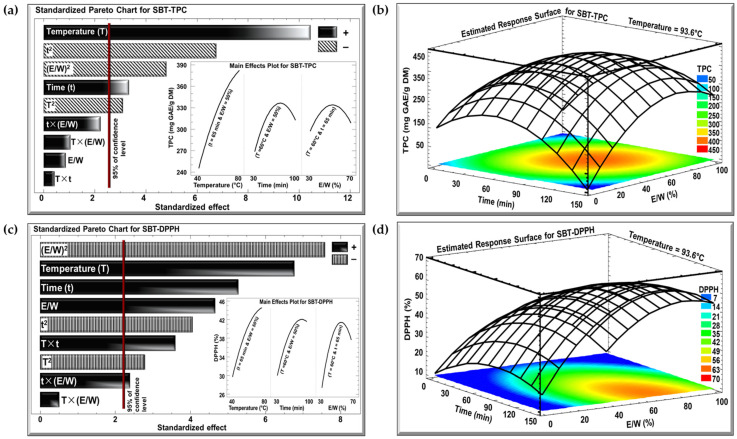
Standardized Pareto charts with inserts for the effect of the studied parameters on (**a**) TPC and (**c**) DPPH inhibition percentage, and estimated response surface for (**b**) TPC and (**d**) DPPH inhibition percentage for SBT extract. (+) indicates a positive effect, and (−) indicates a negative effect.

**Figure 3:**
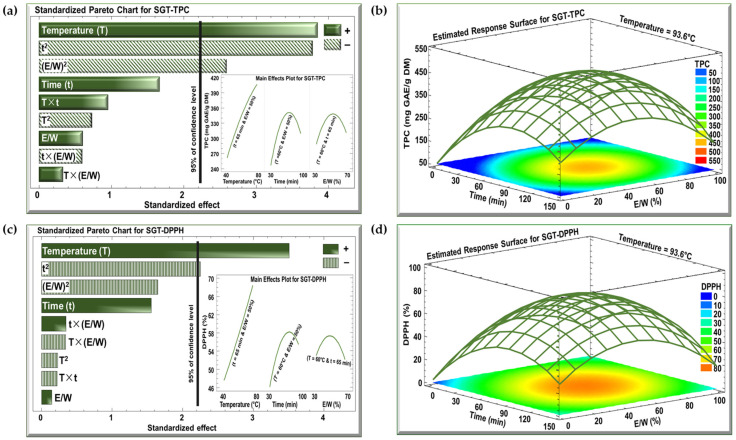
Standardized Pareto charts with inserts for the effect of the studied parameters on (**a**) TPC and (**c**) DPPH inhibition percentage, and estimated response surface for (**b**) TPC and (**d**) DPPH inhibition percentage for SGT extract. (+) indicates a positive effect, and (−) indicates a negative effect.

The effects of the three investigated parameters, temperature (°C), time (min), and E/W ratio (%), on TPC and DPPH were examined using Pareto charts and estimated response surface, as shown in Figure 2 and Figure 3 for the SBT and SGT extracts, respectively. In the Pareto charts, a significant effect with a confidence level greater than 95% is indicated by a vertical bar. For the Pareto chart inserts, one parameter was varied (T from 40 to 80 °C, t from 30 to 100 min, or E/W from 30 to 70%), while the other two parameters were maintained at their central levels (T = 60 °C, t = 65 min, or E/W = 50%).

The Pareto charts in Figure 2a and Figure 3a demonstrate the effect of extraction temperature, time, and the E/W ratio on the TPC for the SBT and SGT extracts, respectively. In both cases, increasing the extraction temperature from 40 to 80 °C had a significant positive linear effect on TPC. For SBT, the TPC rose from 240 to 380 mg GAE/g DM (insert of Figure 2a), while for SGT, it increased from 260 to 410 mg GAE/g DM (insert of Figure 3a), showing a similar temperature-dependent improvement in phenolic extraction. In terms of extraction time, a comparable pattern was observed in both figures. Extending the extraction duration from 30 to 100 min resulted in a significant positive linear effect with a negative quadratic trend. For SBT, the TPC increased from 270 to a peak of 330 mg GAE/g DM before dropping to 310 mg GAE/g DM (insert of Figure 2a). Likewise, in SGT, the TPC rose from 250 to a peak of 350 mg GAE/g DM, and then declined to around 310 mg GAE/g DM (insert of Figure 3a). Regarding the E/W ratio, both extracts showed similar trends in their response to increasing ethanol concentration from 30 to 70%. For SBT, the TPC initially rose from 290 to 330 mg GAE/g DM but then decreased to 310 mg GAE/g DM (insert of Figure 2a). A similar quadratic effect was observed in SGT, where the TPC increased from 290 to 350 mg GAE/g DM before declining back to 310 mg GAE/g DM (insert of Figure 3a).

In the case of the DPPH assay, the Pareto charts (Figure 2c and Figure 3c) revealed trends similar to those observed for TPC. Increasing the extraction temperature from 40 to 80 °C had a significant positive linear effect on DPPH inhibition in both extracts. In SBT, DPPH inhibition rose from 30 to 45% (insert of Figure 2c), while in SGT, it increased from 48 to 69% (insert of Figure 3c). Extending the extraction time from 30 to 100 min also produced a positive linear effect in both extracts. In SBT, the DPPH inhibition increased from 30 to 42%, followed by an insignificant decrease starting around the 80 min mark (insert of Figure 2c). Similarly, in SGT, the inhibition percentage rose from 46 to 58%, followed by a slight decline to 55% due to a negative quadratic effect (insert of Figure 3c). Regarding the E/W ratio, increasing the ethanol content from 30 to 70% had a significant positive effect on DPPH inhibition in SBT, where it increased from 27 to 41%, followed by a negative effect that reduced it to 38% (insert of Figure 2c). In contrast, SGT showed no significant effect for the E/W ratio, with DPPH inhibition ranging from 52% to 55% (insert of Figure 3c).

Increasing the extraction temperature had a significant positive linear effect on both TPC and DPPH yields in SBT and SGT. Several studies have shown that higher extraction temperatures enhance phenolic yield and antioxidant activity [43,44,45]. Elevated temperatures facilitate extraction by softening and perforating plant tissues, increasing polyphenol solubility, and enabling more efficient transfer and extraction [46]. Bindes et al. (2019) reported that the optimal temperature range for the maximum polyphenol yield from green tea leaves is between 60 and 80 °C, after which the yield tends to decline at higher temperatures [47]. Similarly, RSM revealed that increasing the extraction time boosted TPC and DPPH yields up to a certain point, after which a negative effect was observed. This can be attributed to longer exposure times allowing more phenolic compounds with antioxidant properties to diffuse into the solvent [48]. However, extended extraction times, especially at elevated temperatures, may often lead to a reduction in phenolic yield due to compound degradation [49].

Our data indicated that while increasing ethanol concentration initially boosted polyphenol and DPPH yields, a negative quadratic effect emerged at higher concentrations. This observation aligns with previous findings [50,51]. Moreover, several studies have demonstrated that an ethanol concentration of approximately 50% is optimal for extracting polyphenols from tea, yielding the highest antioxidant activity, which further confirms our results [52,53,54].

The optimal extraction conditions for maximizing TPC in both SBT and SGT are shown in Figure 2b and Figure 3b, respectively, while the conditions for maximizing DPPH inhibition are presented in Figure 2d and Figure 3d. The temperature was set at its optimal level of 93.6 °C, while time and the E/W ratio showed significant quadratic effects. Thus, achieving the best extraction results requires an optimal combination of time and E/W ratio. These figures emphasize the interactive effect of time and E/W ratio on extraction efficiency at 93.6 °C. As shown in the orange- and red-shaded areas of these figures, the maximum TPC for SBT reached approximately 400 mg GAE/g DM (Figure 2b), whereas SGT achieved a higher TPC of around 450 mg GAE/g DM under similar conditions (Figure 3b). Likewise, shown within the orange- and red-shaded areas, the maximum DPPH inhibition for SBT was around 50% (Figure 2d), while SGT demonstrated a higher inhibition percentage, reaching approximately 80% (Figure 3d).

Table 2 presents the second-degree regression model equations used to predict the response values based on statistical analysis.

### 3.2. Optimization of Extraction

**Figure 4:**
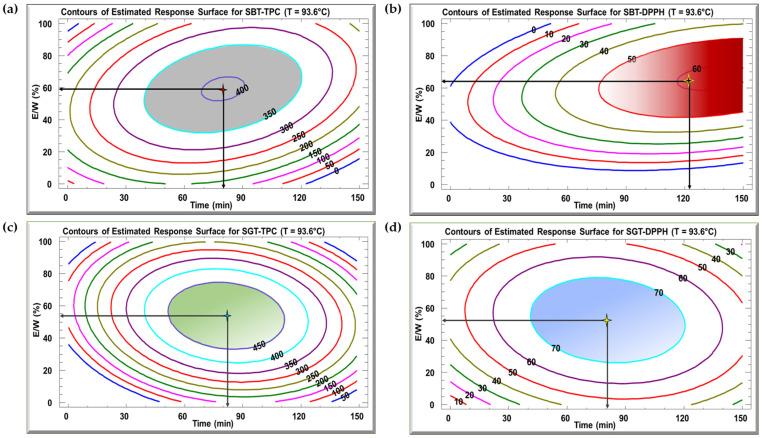
Contour plots of the estimated response surface for TPC (**a**,**c**) and DPPH (**b**,**d**) as a function of time and the ethanol/water ratio for SBT and SGT extracts, respectively.

Figure 4 displays the contour plots of the estimated response surface for TPC (Figure 4a,c) and DPPH inhibition (Figure 4b,d) as a function of time and E/W ratio at 93.6 °C for SBT and SGT, respectively. These plots show that a given TPC or DPPH value can be achieved through different combinations of time and E/W ratio at a fixed temperature of 93.6 °C. For the SBT extract, the optimal conditions for maximizing TPC (404 mg GAE/g DM) were a time of 79.9 min and an E/W ratio of 59.4% (Figure 4a). In terms of DPPH inhibition, the ideal conditions to reach 60.8% inhibition were 123.9 min at a 65% E/W ratio (Figure 4b). For SGT extract, the maximum yield of 452 mg GAE/g DM was observed when the extraction was carried out for 82.1 min and a 53.8% E/W ratio (Figure 4c). Regarding DPPH inhibition, the highest value of 78.3% was recorded at an extraction time of 81 min and a 52.6% E/W ratio (Figure 4d).

**Figure 5:**
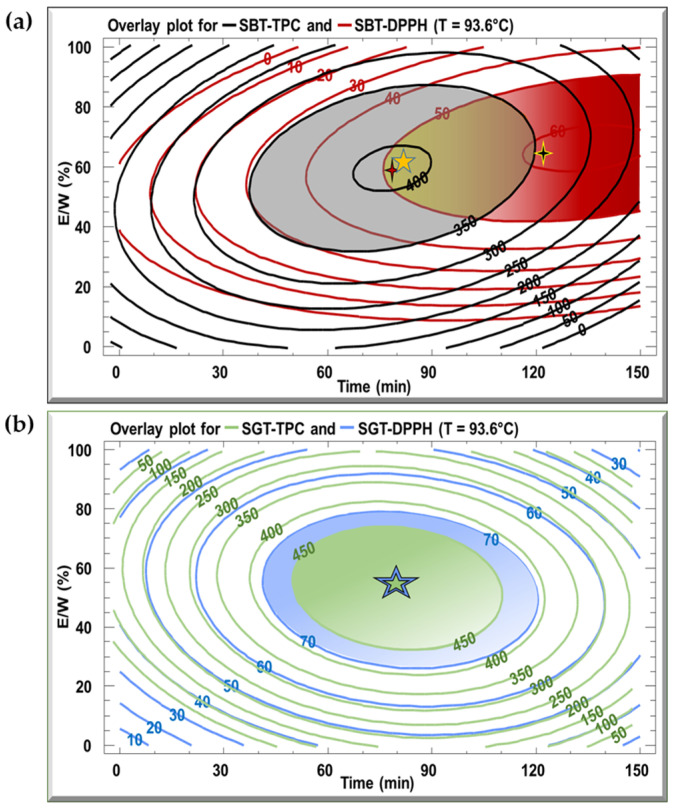
Overlay plots generated from contours of estimated response surface for TPC and DPPH for (**a**) SBT and (**b**) SGT extracts.

The optimal extraction conditions for the SBT and SGT extracts are outlined in Table 3, with the model demonstrating satisfactory adequacy, as indicated by high R^2^ values ranging from 70.2% to 94.9%. Multiple response optimization was employed to simultaneously maximize polyphenol content and antioxidant activity. Figure 5 displays overlay plots, with blue lines representing TPC and red lines for DPPH inhibition, for both extracts. The optimal conditions for SBT, yielding the highest TPC (404 mg GAE/g DM) and DPPH inhibition (51.5%), are found in the overlapping region, corresponding to a temperature of 93.6 °C, extraction time of 79.9 min, and an E/W ratio of 59.4% (Figure 5a). For SGT, the optimal extraction parameters, located in the overlapping zones of Figure 5b, are 93.6 °C, 81.7 min, and an E/W ratio of 53.2%, resulting in a TPC of 452 mg GAE/g DM and 78.3% DPPH inhibition.

To validate the model’s predicted optimal conditions, spent tea leaves were extracted according to the parameters outlined in Table 3. The resulting TPC and DPPH inhibition values for both the SBT and SGT extracts closely matched the predicted outcomes, confirming the accuracy of the model’s estimations. Under optimal extraction conditions, SGT (452 mg GAE/g DM) exhibited a higher TPC compared to SBT (404 mg GAE/g DM). This difference can be attributed to the fermentation process involved in black tea production, which is known to cause significant polyphenol losses, thereby reducing the total phenolic content in the resulting extract [3,55]. In comparison, studies on other waste byproducts yielded lower TPC values. For instance, potato peels extracted using WB (90 °C for 70 min) and infrared (IR) (80 °C for 10 min) resulted in a polyphenol content of 3.5 mg GAE/g DM [56]. Olive leaves extracted by WB and IR methods showed TPC values of 27.12 and 36.23 mg GAE/g DM, respectively [57]. Unmilled and milled grape pomace had TPC values of 1.1 and 1.4 mg GAE/g DM, respectively, while grape seed extracts from red and white varieties contained 301 and 206 mg GAE/g DM [58]. Sesame seed coats yielded TPC values of 7.3 and 7.8 mg GAE/g DM when extracted by WB and IR, respectively [44]. The phenolic content of date seeds varied by variety, with Kabkab exhibiting the highest TPC (271 mg GAE/g DM) and Majdool the lowest (63 mg GAE/g DM) [59]. Other waste byproducts such as apricot pomace [60], grapefruit peels [61], and pomegranate peels [62] had TPC values of 10 mg GAE/g DM, 86 mg GAE/g DM, and 46 mg GAE/g DM, respectively, under various extraction conditions. Even for tea leaves obtained to mimic tea prepared for consumption (green, black, oolong, white, yellow, and dark teas), TPC values ranged from 24.8 to 253 mg GAE/g DM [63]. These comparisons highlight the exceptional polyphenol content of spent tea leaves, making them a particularly rich source of phenolic compounds relative to other agricultural waste byproducts.

Due to the strong correlation between TPC and antioxidant activity, the higher phenolic content in SGT resulted in greater DPPH inhibition (78.3%) compared to SBT (51.5%) [64,65]. Notably, the DPPH inhibition percentages observed for spent tea leave extracts in this study fall within the range reported for tea extracts (2.60–95.42%), as determined using the DPPH radical method [66]. Furthermore, studies have demonstrated comparable antioxidant activities for SBT extracts. For example, antioxidant activity equivalent to 64.20 g gallic acid equivalents/kg SBT was achieved with ethanol–water (50% *w*/*w*) at 125 °C and 0.3 MPa [30], while extraction at 180 °C with 71% ethanol yielded 69.08 mg gallic acid equivalents/g SBT [32]. Another study, using a preparation of 50 g of black tea leaves brewed in 1000 mL of hot water (100 °C) for five minutes followed by filtration and drying, reported 57.83% DPPH inhibition [67]. These findings collectively highlight the antioxidant potential of spent tea leaves, as supported by results across different extraction methods and conditions.

### 3.3. Antibacterial Activity of Spent Tea Extracts

Following the optimization of polyphenol extraction for SBT and SGT, the antibacterial activities of the extracts were evaluated under optimal conditions using the disk diffusion assay (Supplementary Material, Figures S2 and S3). The diameters of the inhibition zones (in mm) obtained from the assay are presented in Table 4. The results indicated that both tea extracts demonstrated antibacterial activity against Gram-positive bacteria, specifically *S. aureus* and *B. subtilis*. Notably, SGT exhibited a stronger effect against *S. aureus* than SBT, with inhibition zones measuring 17 ± 0.1 mm and 10 ± 0.2 mm, respectively. Similarly, SGT demonstrated slightly greater efficacy in inhibiting *B. subtilis*, with inhibition zones of 14 ± 0.1 mm compared to 13 ± 0.4 mm for SBT. Treatment with gentamicin resulted in a 32 ± 0.2 mm inhibition zone. Our findings confirm the antibacterial efficacy of SBT and SGT extracts against Gram-positive bacteria, aligning with previous studies [16,27,68]. The higher phenolic content in SGT (423 mg GAE/g DM) may contribute to its enhanced antibacterial effect, as indicated by the larger zones of inhibition compared to SBT (382 mg GAE/g DM). Overall, increased fermentation of tea tends to lower its antibacterial activity, suggesting that green tea has a greater efficacy compared to black tea [28,29].

On the other hand, no inhibition zones were observed against the Gram-negative bacteria (*E. coli* and *S. typhimurium*) (Table 4). This is consistent with findings from a study where tea extracts were ineffective against *E. coli* but showed activity against *S. aureus* [66]. Generally, plant extracts exhibit greater antibacterial activity against Gram-positive bacteria than Gram-negative bacteria [69,70]. The outer membrane of Gram-negative bacteria contains a lipopolysaccharide layer that acts as a barrier, hindering the interaction of polyphenols with the peptidoglycan layer and contributing to bacterial resistance [71].

### 3.4. High-Performance Liquid Chromatography (HPLC) Analysis of SBT and SGT Extracts

HPLC analysis was conducted to identify and quantify the phenolic compounds in the SBT and SGT extracts, with the results summarized in Table 5. Phenolic compounds were identified based on their retention times, and their concentrations were estimated from the peak areas. In the SBT extract, hydroxybenzoic acid was the most abundant compound (360.7 mg/L), followed by caffeic acid (13.02 mg/L), quercetin (8.62 ± 0.10 mg/L), chlorogenic acid (2.79 ± 0.23 mg/L), and *p*-coumaric acid (0.15 ± 0.01 mg/L). For the SGT extract, rutin was the predominant compound (42.73 mg/L), with ellagic acid (7.80 mg/L) and caffeic acid (6.14 mg/L) also identified. The phenolic compounds identified and quantified through HPLC analysis were generally consistent with findings from previous studies, where most of the detected compounds have been reported before [4,33]. However, unlike earlier reports, our analysis did not detect gallic acid, catechin, protocatechuic acid, or trans-cinnamic acid. These differences may be explained by variations in extraction parameters (such as time, temperature, solvent type, and solid-to-solvent ratio), as well as factors like harvest season, tea cultivars, cultivation conditions, and the degree of fermentation [72,73,74]. In future studies, HPLC coupled with tandem mass spectrometry will be utilized to broaden the range of detected compounds.

## 4. Conclusions

This study successfully demonstrated that spent tea leaves, waste generated from tea consumption, could be transformed into a valuable source of bioactive compounds, particularly phenolics, through optimized extraction. The RSM model was developed by adjusting temperature, extraction time, and the E/W ratio, while keeping the solid-to-solvent ratio at 1/25 (g/mL) for SBT and 1/20 (g/mL) for SGT. The SBT extract resulted in a TPC of 404 mg GAE/g DM and a DPPH inhibition of 51.5% corresponding to an extraction temperature of 93.6 °C, a time of 79.9 min, and an E/W ratio of 59.4%. On the other hand, the SGT extract resulted in a TPC of 452 mg GAE/g DM and 78.3% DPPH inhibition at 93.6 °C, 81.7 min, and an E/W ratio of 53.2%. The observed differences are likely due to the fermentation process in black tea production, which significantly reduces polyphenol content in the final extract. Additionally, both extracts showed antibacterial properties, particularly against Gram-positive bacteria. Determining the minimum inhibitory concentration (MIC) of the extracts in future research will provide a quantitative assessment of their antibacterial efficacy. The phytochemical analysis revealed that hydroxybenzoic acid was the main phenolic compound detected in SBT at 360.7 mg/L, while rutin was the highest compound identified in SGT at 42.73 mg/L, further emphasizing the distinct composition and potential health benefits of each extract. Future studies will expand the analysis of bioactive compounds by identifying additional phenolic compounds using HPLC, enabling a more detailed characterization of the extracts.

This study was conducted using spent leaves from one black tea and one green tea variety, which limits the generalizability of the findings to other tea origins and types. These findings suggest that spent tea leaves, often regarded as waste, could be effectively repurposed as a source of valuable bioactive compounds with antioxidant and antibacterial properties. This study not only highlights the implications for sustainable waste management and the development of functional ingredients for use in the food, pharmaceutical, and cosmetic industries but also supports the move towards a circular economy by utilizing waste from tea consumption, offering an eco-friendly and cost-effective strategy for the valorization of by-products.

## Figures and Tables

**Table 1:** Central composite design for independent variables and their responses as TPC (mg GAE/g DM) and DPPH (%).

Run	Variables [Coded Values]			Responses			
	Temperature (°C)	Time (min)	E/W Ratio (%)	SBT		SGT	
				TPC (mg GAE/g DM)	DPPH (%)	TPC (mg GAE/g DM)	DPPH (%)
1	40 [−1]	30 [−1]	30 [−1]	160	17.4	120	29.1
2	80 [+1]	30 [−1]	30 [−1]	274	14.5	164	41
3	40 [−1]	100 [+1]	30 [−1]	151	8.7	149	35.9
4	80 [+1]	100 [+1]	30 [−1]	238	30.8	266	47.9
5	40 [−1]	30 [−1]	70 [+1]	173	20.4	163	36.2
6	80 [+1]	30 [−1]	70 [+1]	281	24.8	219	50
7	40 [−1]	100 [+1]	70 [+1]	199	29.8	113	34.1
8	80 [+1]	100 [+1]	70 [+1]	363	50.4	283	54.8
9	26.3 [−α]	65 [0]	50 [0]	153	14.9	176	42.1
10	93.6 [+α]	65 [0]	50 [0]	405	48.9	435	77.2
11	60 [0]	6.1 [−α]	50 [0]	155	17.2	113	32.9
12	60 [0]	123.9 [+α]	50 [0]	296	38.9	282	60.7
13	60 [0]	65 [0]	16.3 [−α]	295	12.7	237	59.9
14	60 [0]	65 [0]	83.6 [+α]	227	22.1	281	43.7
15	60 [0]	65 [0]	50 [0]	368	38.6	346	54
16	60 [0]	65 [0]	50 [0]	380	39.3	284	60.5
17	60 [0]	65 [0]	50 [0]	390	42.1	300	52.3
18	60 [0]	65 [0]	50 [0]	319	44	288	59.5
19	60 [0]	65 [0]	50 [0]	346	37.8	310	54.4
20	60 [0]	65 [0]	50 [0]	340	38.9	293	60.9

**Table 2:** Second-order regression equations for SBT and SGT extracts.

Extract	Equations
SBT	TPC = −272 + 8 × T + 3.6 × t + 4.8 × E/W − 0.05 × T^2^ + 0.005 × T × t + 0.022 × T × E/W − 0.035 × t^2^ + 0.027 × t × E/W − 0.077 × E/W^2^
	DPPH = −48 + 0.69 × T − 0.06 × t + 1.85 × E/W − 0.007 × T^2^ + 0.007 × T × t + 0.002 × T × E/W − 0.004 × t^2^ + 0.005 × t × E/W − 0.02 × E/W^2^
SGT	TPC = −491 + 4.46 × T + 7.3 × t + 12.6 × E/W − 0.033 × T^2^ + 0.033 × T × t + 0.02 × T × E/W − 0.057 × t^2^ − 0.021 × t × E/W − 0.12 × E/W^2^
	DPPH = −19.3 + 0.075 × T + 0.85 × t + 1.1 × E/W + 0.0017 × T^2^ + 0.001 × T × t + 0.003 × T × E/W − 0.005 × t^2^ − 0.002 × t × E/W − 0.012 × E/W^2^

**Table 3:** Optimum extraction conditions for SBT and SGT extracts.

Parameters	Optimum Conditions			
	SBT		SGT	
	TPC	DPPH	TPC	DPPH
Temperature (°C)	93.6	93.6	93.6	93.6
Time (min)	79.9	123.9	82.1	80.8
E/W ratio (%)	59.4	65	53.8	52.6
TPC predicted (mg GAE/g DM)	404	-	452	-
DPPH predicted (%)	-	60.8	-	78.3
Model’s R-squared	86.4	94.9	84.4	70.2
**Parameters**	**Multiple Optimization**			
	**SBT**		**SGT**	
Temperature (°C)	93.6		93.6	
Time (min)	79.9		81.7	
E/W ratio (%)	59.4		53.2	
TPC predicted (mg GAE/g DM)	404		452	
TPC observed (mg GAE/g DM)	383		423	
DPPH predicted (%)	51.5		78.3	
DPPH observed (%)	50.2		76.4	

**Table 4:** Antibacterial activity of SBT and SGT against *Staphylococcus aureus*, *Bacillus subtilis*, *Salmonella Typhimurium*, and *Escherichia coli*.

Bacterial Strain	Zone of Inhibition (mm, Mean ± SD)	
	SBT	SGT
*Staphylococcus aureus*	10 ± 0.2 mm	17 ± 0.1 mm
*Bacillus subtilis*	13 ± 0.4 mm	14 ± 0.1 mm

**Table 5:** Phenolic compounds identified in SBT and SGT extracts by HPLC.

Phenolic Compound	Concentration (mg/L)	
	SBT	SGT
Rutin	-	42.73 ± 0.00
Caffeic acid	13.02 ± 0.00	6.14 ± 0.00
Hydroxybenzoic acid	360.7 ± 0.00	-
Ellagic acid	-	7.80 ± 0.00
Chlorogenic acid	2.79 ± 0.23	-
*p*-coumaric acid	0.15 ± 0.01	-
Quercetin	8.62 ± 0.10	-

## Data Availability

Data are contained within the article.

## Supplementary Materials

The following supporting information can be downloaded at https://www.mdpi.com/article/10.3390/antiox13121588/s1: Figure S1: Strainer and presser used in the preparation of spent tea extracts. Figure S2: Effect of SBT (a,b) and SGT (c,d) against Gram-positive bacterial strains: *S. aureus* and *B. subtilis*. Figure S3: Effect of SBT (a,b) and SGT (c,d) against Gram-negative bacterial strains: *S. Typhimurium* and *E. coli.*
