# Supplementary material for: From Spent Black and Green Tea to Potential Health Boosters: Optimization of Polyphenol Extraction and Assessment of Their Antioxidant and Antibacterial Activities
Source: Antioxidants (Basel). 2024 Dec 23;13(12):1588. doi: 10.3390/antiox13121588 (PMC11673901; doi:10.3390/antiox13121588)
Supplement: Supplementary file 1 [file antioxidants-13-01588-s001.zip › antioxidants-3349963-supplementary.pdf]

Supplementary Materials

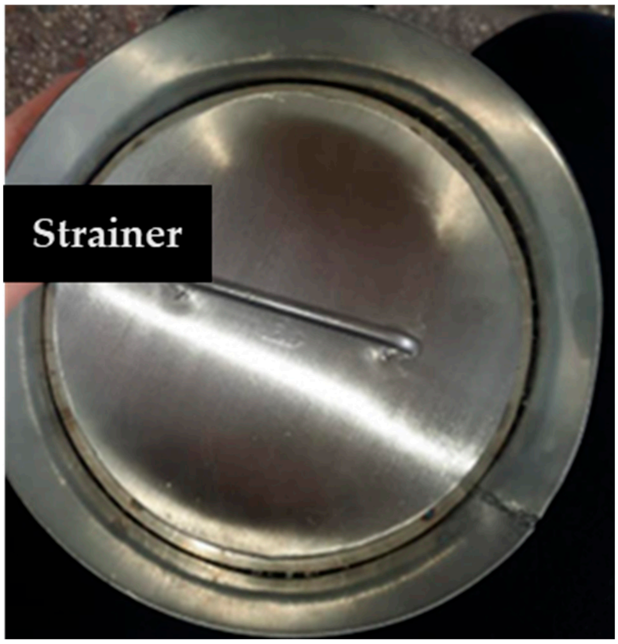

Figure S1: Strainer and presser used in the preparation of spent tea extracts.

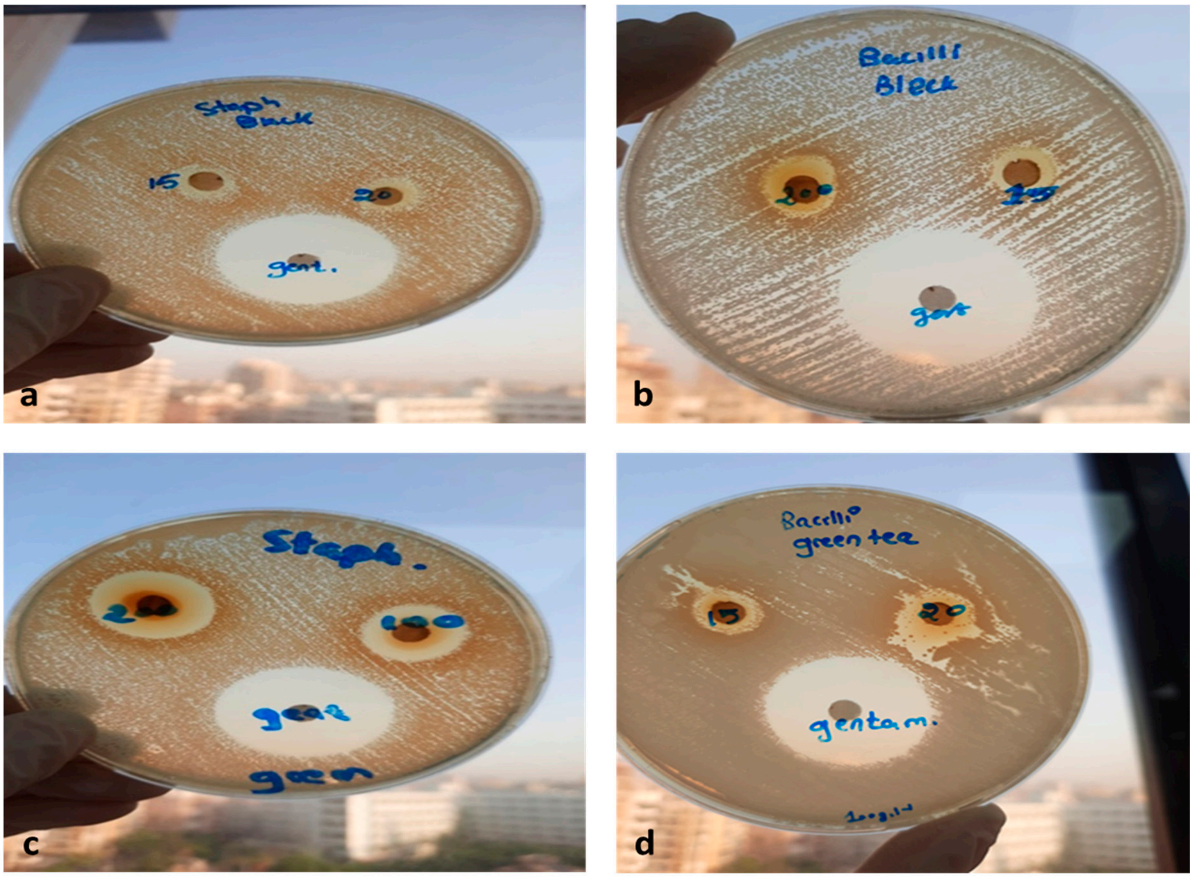

Figure S2: Effect of SBT (a,b) and SGT (c,d) against Gram-positive bacterial strains: *S. aureus* and *B. subtilis.*

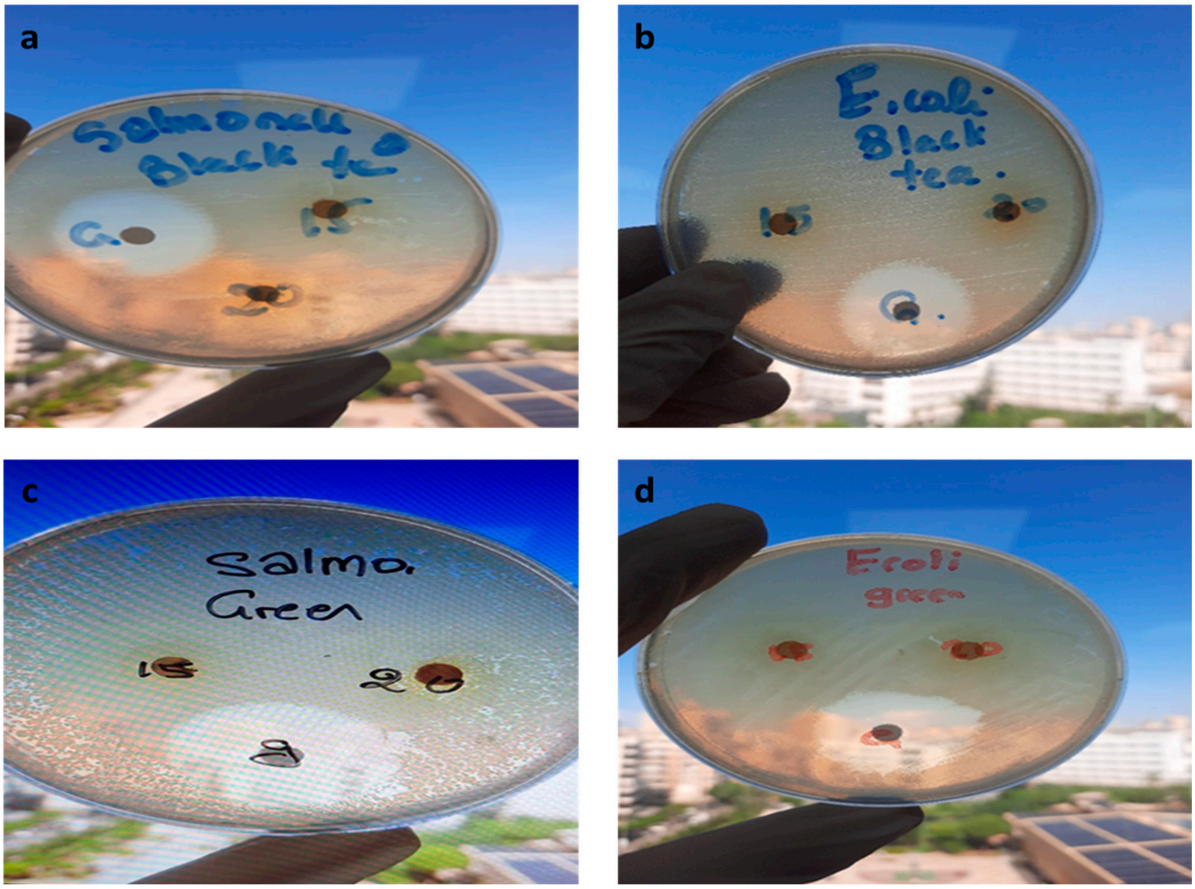

Figure S3: Effect of SBT (a,b) and SGT (c,d) against Gram-negative bacterial strains: *S.* Typhimurium and *E. coli.*
